# *Arecae pericarpium* water extract alleviates chronic pancreatitis by deactivating pancreatic stellate cells

**DOI:** 10.3389/fphar.2022.941955

**Published:** 2022-08-29

**Authors:** Bitna Kweon, Dong-Uk Kim, Jin-Young Oh, Hyuncheol Oh, Youn-Chul Kim, Yeun-Ja Mun, Gi-Sang Bae, Sung-Joo Park

**Affiliations:** ^1^ Department of Pharmacology, School of Korean Medicine, Wonkwang University, Iksan, Jeollabuk-do, South Korea; ^2^ Hanbang Cardio-Renal Syndrome Research Center, School of Korean Medicine, Wonkwang University, Iksan, Jeollabuk-do, South Korea; ^3^ Institute of Pharmaceutical Research and Development, College of Pharmacy, Wonkwang University, Iksan, Jeollabuk-do, South Korea; ^4^ Department of Anatomy, College of Korean Medicine, Wonkwang University, Iksan, Jeollabuk-do, South Korea; ^5^ Research Center of Traditional Korean Medicine, Wonkwang University, Iksan, Jeollabuk-do, South Korea; ^6^ Department of Herbology, School of Korean Medicine, Wonkwang University, Iksan, Jeollabuk-do, South Korea

**Keywords:** chronic pancreatitis (CP), *Arecae pericarpium* (ARP), fibrosis, cerulein, pancreatic stellate cells (PSCs)

## Abstract

Chronic pancreatitis (CP) is a chronic inflammatory disease of the pancreas with irreversible morphological changes. *Arecae pericarpium* (ARP), known to improve gastrointestinal disorders, has not yet been reported to inhibit fibrosis in CP. Therefore, we investigated the beneficial effects of ARP on cerulein-induced CP. Cerulein (50 μg/kg) was administered intraperitoneally to mice every hour, six times a day, four times a week for a total of 3 weeks to induce CP. To ascertain the prophylactic effects of ARP, ARP water extract (50, 100, or 200 mg/kg) or saline was administered intraperitoneally 1 h before the onset of CP. To determine the therapeutic effects of ARP, ARP water extract (200 mg/kg) or saline was administered for a total of 1 week or 2 weeks, starting 2 weeks or 1 week after the onset of CP. The pancreas was collected immediately for histological analysis. Additionally, to determine the effectiveness and mechanism of ARP in alleviating pancreatic fibrosis, pancreatic stellate cells (PSCs) were isolated. ARP treatment considerably improved glandular atrophy and inflammation and repressed collagen deposition in the pancreas. Furthermore, ARP water extract inhibited extracellular matrix (ECM) constituents such as alpha-smooth muscle actin (α-SMA), collagen I, and fibronectin 1 (FN1) in pancreatic tissue and PSCs. ARP also suppressed transforming growth factor-β (TGF-β) signaling by inhibiting Smad2 phosphorylation. Our study suggests that ARP exhibits anti-fibrotic effects in cerulein-induced CP by inhibiting TGF-β/Smad signaling.

## Introduction

Chronic pancreatitis (CP) is a chronic inflammatory disease of the pancreas characterized by morphological changes such as abnormal, progressive, and irreversible fibrosis and atrophy in granular tissue, resulting in the malfunction of the pancreatic exocrine and endocrine systems (Apte et al., 2011; Brock et al., 2013; Gardner et al., 2020). Typical clinical features of CP include abdominal pain, indigestion, and diabetes, which damage the overall quality of life, thereby impeding social life (Drewes, 2013). In addition to environmental factors such as alcohol and smoking, genetic factors are also crucial for triggering CP, thereby requiring utmost attention even at normal times (Gardner et al., 2020; Hegyi et al., 2020). The overall prevalence rate of CP is estimated to be 40–50 per 100,000 population and it is increasing gradually (Machicado and Yadav, 2017; Oh and Song, 2020). Moreover, recent studies have reported that the patients with CP are at higher risk for other diseases such as cancers and diabetes than individuals without CP (Bang et al., 2014; Oh and Song, 2020; Han et al., 2022). Therefore, treating CP at the initial stage is necessary to ensure a favorable prognosis.

Pancreatic stellate cells (PSCs) play a vital role in the initiation of CP (Wells and Crawford, 1998). During the development of CP, a wide range of signals, such as cytokines and transcription factors, activate PSCs that are normally in the quiescent state (Jin et al., 2020). Once stimulated, PSCs are divided into myofibroblast-like phenotypes and secrete diverse extracellular matrix (ECM)-related factors. Platelet-derived growth factor (PDGF) is a principal factor synthesized by inflammatory cells; it augments PSC proliferation and migration (Wells and Crawford, 1998; Jin et al., 2020). Transforming growth factor-β (TGF-β) is a multifunctional cytokine that regulates cell proliferation, differentiation, cell death, immune cell function, and ECM production (Roberts and Sporn, 1985; Massague, 1998; Flanders, 2004). TGF-β is a potent fibrosis mediator whose expression increases in the vicinity of impaired cells. It increases the expression of alpha-smooth muscle actin (α-SMA) and is involved in collagen and fibronectin 1 (FN1) synthesis by stimulating PSCs (Apte et al., 1999; Haber et al., 1999; Jin et al., 2020). The Smad2/3 cascade is known to play an important role in the TGF-β-induced deposition of fibrous factors, such as α-SMA, collagen, and FN1 induced by TGF-β (Dobaczewski et al., 2010). They are phosphorylated directly following their interaction with TGF-β receptor I upon TGF-β stimulation (Zhang et al., 1996; Nakao et al., 1997). Thus, inhibition of TGF-β/Smad signaling could be an important factor in regulating PSC activation.

Arecae pericarpium (ARP) is a medicinal herb classified as a qi-regulating herb in the palm family. It has been used to treat constipation, abdominal distension, and edema in East Asia (Tey et al., 2021). Recently, ARP has been reported to be effective in treating gastrointestinal disorders by improving gastrointestinal mobility and controlling intestinal transmission (Wang et al., 2004). Although ARP is considered a relatively safe drug due to fewer side effects and the infrequent incidence of allergic reactions, its pharmacological efficacy has rarely been studied (Xu et al., 2002).

As far as we know for now, there has been no research on the effect of ARP water extract on the fibrosis regulation as well as pancreatitis. Therefore, we would like to first identify the anti-fibrotic effect of the ARP water extract in CP. The main purpose of this study was to investigate the anti-fibrotic effects and related mechanisms of the ARP water extract on CP. The pancreas weight/body weight (PW/BW) ratio, the morphological appearance of the pancreas, and the levels of fibrosis-related genes were evaluated to investigate the protective or therapeutic effects of ARP on cerulein-induced CP.

## Materials and methods

### Chemicals and Reagents

Dulbecco’s Modified Eagle Medium (DMEM), high glucose, pyruvate (11,995-065), Fatal Bovine Serum (FBS) (16,000-044), Penicillin/streptomycin (15140-122) and 2-mercaptoethanol (21985-023) were purchased from Thermo Fisher Scientific (Waltham, MA, United States). Surgipath Paraplast Paraffin Wax (39601006) and FSC 22 Frozen Section Media (3801480) were purchased from Leica Biosystems (Chicago, IL, United States). SIS3 (S0447), Phosphotase inhibitor cocktail (p5726), and Glycerol (G6279) were purchased from Sigma-Aldrich Chemical Co. (St. Louis, MO, United States). Tris-HCl Buffer (pH 6.8) (T8102-050) and Tween 20 (T9100-010) were purchased from GenDEPOT (Katy, TX, United States). Radioimmunoprecipitation assay (RIPA) Buffer (IBS-BR004) was purchased from iNtRON Biotechnology (Sungnam, Republic of Korea). EZ block Protease inhibitor cocktail (K272-1) was purchased from Biovision (Milpitas, CA, United States).

### Plant materials

ARP was obtained from Kwang Myung Dang Medical Herbs (Ulsan, South Korea). ARP extract was prepared by decocting dried ARP (100 g) with distilled water (1 L). The decoction was boiled for 150 min. The aqueous extract was freeze-dried to produce a powder with a yield of 11.2 g. The resulting powder was dissolved in distilled water and filtered. Filtrates were stored at 4°C until use.

### Animal models

All experiments were performed according to protocols approved by the Animal Care Committee of Wonkwang University. C57BL/6 mice (six to eight weeks old, female, weighing 15–20 g) were purchased from Samtako Biokorea Co. Ltd. (Osan, KyungKiDo, South Korea). Mice were allowed to breed and were housed in standard shoebox cages in a climate-controlled room maintained at an ambient temperature of 23°C ± 2°C and a 12 h light–dark cycle for 7 days. They were fed standard laboratory chow, provided with water *ad libitum*, and randomly assigned to the control and experimental groups. CO_2_ inhalation was used for anesthesia and euthanasia.

### Experimental design

**FIGURE 1:**
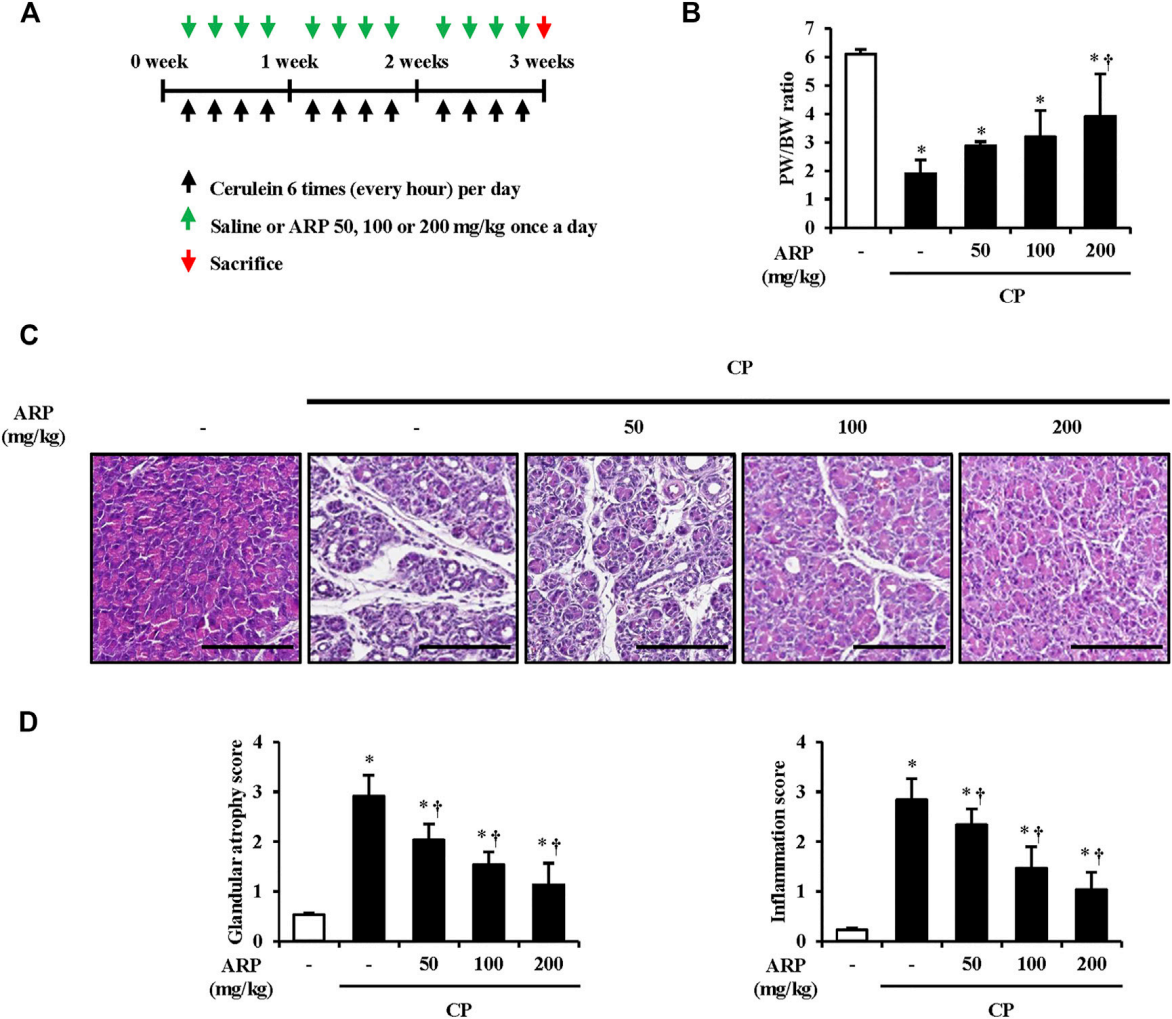
Effects of ARP water extract on pancreas morphological change during cerulein-induced CP. **(A)** Scheme of CP model in mice to examine the prophylactic effect of ARP water extract. **(B)** Pancreatic weight/body weight ratio in the saline, CP, and ARP groups (50, 100, and 200 mg/kg). **(C)** Representative Hematoxylin and Eosin-stained sections of the pancreas. **(D)** Pathological scores of pancreatic tissues. Data are represented as means ± SEM (*n* = 3 per group). Results are representative of three experiments. *p < 0.05 vs saline alone; ^†^ *p* < 0.05 vs. CP. Original magnification, ×400. Scale bar = 50 μm.

Mice were intraperitoneally injected with cerulein 50 μg/kg (H-3220; Bachem AG, Switzerland) six times per day at 1 h intervals, four times per week, for 3 weeks. In the pre-treatment groups, mice were intraperitoneally injected with ARP water extract (50, 100, or 200 mg/kg) or saline (control group) 1 h before the first injection. In the post-treatment group, ARP water extract (200 mg/kg) was administered 2 weeks or 1 week after the first cerulein injection. Control groups were intraperitoneally administered with ARP (200 mg/kg) or saline under the same conditions. After 3 weeks, the mice were sacrificed, and the pancreas was rapidly removed and stored at −80°C for further study (Figure 1A). These results were confirmed by conducting additional triplicate experiments.

### Histological analysis

After the mice were sacrificed, the pancreas tissue was washed with ice-cold stroke-physiological saline solution and fixed in 10% neutral-buffered-formalin solution (HT-501128; Sigma-Aldrich Chemical Co., St. Louis, MO, United States) for 24 h. After routine processing such as alcohol dehydration and xylene permeabilization, the tissues were embedded in paraffin, sectioned to 4 µm thickness, and stained with hematoxylin for 8 min and eosin for 2 min. The samples were scored on a scale from 0 to 3 (0 corresponding to normal appearance and three corresponding to severe disease) based on the presence of inflammation and glandular atrophy.

### Biochemical analysis

ARP was intraperitoneally administered to mice (*n* = 3 per group). After 24 h of administration, serum was obtained and separated by centrifugation maintained at 2339× g for 5 min. Fresh serum was used to analyze the alkaline phosphatase (ALP), alanine transaminase (ALT), aspartate transaminase (AST), blood urea nitrogen (BUN), and creatinine (CREA) levels using biochemical kits (NX700i; Fujifilm, Japan).

### Masson’s trichrome staining

MT staining was performed to assess collagen deposition. Deparaffinized and rehydrated slides were stained using an Masson’s trichrome (MT) stain kit (25,088; Polysciences, Warrington, Pennsylvania, United States) according to the manufacturer’s instructions. The relative intensity was measured using the Leica microscopy software (Wetzlar, Germany).

### Immunofluorescence staining

Immunofluorescence analysis of α-smooth muscle actin (α-SMA) and collagen I was performed using the pancreatic tissue. The pancreatic tissues were fixed in a 10% neutral-buffered-formalin solution for 24 h, embedded within optimal cutting temperature compound, and cut into 9 μm sections. The tissues were incubated with primary antibodies against α-SMA (1:500; sc-32251; Santa Cruz Biotechnology, Texas, Dallas, United States) and collagen I (1:250; ab34710; Abcam, Cambridge, United Kingdom) overnight at 4°C, followed by fluorescence-labeled secondary antibodies Alexa Fluor^®^ 594 goat anti-mouse (1:2,000; A11032; Invitrogen, Thermo Fisher Scientific, Waltham, MA, United States) and Alexa Fluor^®^ 488 goat anti-rabbit (1:2,000; A27034; Invitrogen, Thermo Fisher Scientific, Waltham, MA, United States) at RT for 2 h. Nuclei were counter-stained with 4′,6-diamidino-2-phenylindole (DAPI; 5 ng/ml; D1306; Invitrogen, Thermo Fisher Scientific, Waltham, MA, United States) at room temperature for 5 min. The stained sections were visualized using a confocal laser microscope (Olympus Corporation, Tokyo, Japan).

### Real-time RT-PCR

Total RNA was isolated from the pancreas or PSCs using an Easy-BlueTM RNA extraction kit (17061; iNtRON Biotechnology, Sungnam, Republic of Korea). RNA purity was confirmed using the Gene Quant Pro RNA calculator (Biochrom, Inc., Cambridge, United Kingdom). Reverse transcription of RNA to cDNA was performed using an ABI cDNA synthesis kit (4,387,406; Applied Biosystems, Thermo Fisher Scientific, Waltham, MA, United States). Subsequently, the cDNA was amplified using the SYBR Premix kit (4,367,659; Applied Biosystems, MA, United States) using a StepOne Plus Real-Time PCR system (Applied Biosystems, MA, USA). The primer sequences used in this experiment were as follows: Acta-2: forward (5′-GTC CCA GAC ATC AGG GAG TAA-3′) and reverse (5′-TCG GAT ACT TCA GCG TCA GGA-3′); FN1: forward (5′-GAT GTC CGA ACA GCT ATT TAC CA-3′) and reverse (5′-CCT TGC GAC TTC AGC CAC T -3′); Collagen Ⅰ: forward (5′-GTG GTG ACA AGG GTG AGA CA-3′) and reverse (5′-GAG AAC CAG GAG AAC CAG GA-3′); PPP2R2A: forward (5′- CCG TGG AGA CAT ACC AGG TA -3′) and reverse (5′- AAC ACT GTC AGA CCC ATT CC -3′) and glyceraldehyde 3-phosphate dehydrogenase (GAPDH): forward (5′-TCC CAC TCT TCC ACC TTC GA-3′) and reverse (5′-AGT TGG GAT AGG GCC TCT CTT G-3′). The amplification conditions were as follows: 30 s at 95°C, 40 cycles at 95°C for 5 s and 60°C for 60 s each, dissociation for 15 s at 95°C and 30 s at 60 °C, and then 15 s at 95°C on ABI Step one Plus. StepOne software (Applied Biosystems, MA, United States) was used for data analysis. Relative gene expression (target gene expression normalized to that of the endogenous control gene) was calculated using the comparative Ct method (2–ΔΔCt). The analysis was independently conducted three times. Melting curves for GAPDH, Acta-2, Collagen Ⅰ, FN1 and PPP2R2A are in the Supplementary Figure S1.

### Pancreatic stellate cells isolation

PSCs were isolated from C57BL/6 mice via digestion of the pancreatic tissue and Nycodenz density gradient centrifugation as described previously (Zang et al., 2015). Briefly, the pancreas of C57BL/6 mice was removed, chopped with scissors, and digested with collagenase containing DMEM/high glucose supplemented with 10% FBS and 1% penicillin/streptomycin (1 mg/5 ml) for 15 min at 37°C in a shaking water bath. After collagenase digestion, the cell suspension was filtered through a 100 μm nylon mesh (352360; Falcon Life Sciences Ltd., United Kingdom) and subjected to density gradient centrifugation using Nycodenz. Subsequently, the cells were collected from the top of the gradient, washed twice, resuspended in DMEM/high glucose supplemented with 10% FBS and 1% penicillin/streptomycin, and incubated in an environment with 95% O_2_ and 5% CO_2_. All experiments were performed by culturing cells between passages 0 and 3. After confirming that ARP did not exhibit any cytotoxic effects on PSCs at the specified concentrations, we continued with the following experiments.

### Cell treatment

To assess mRNA levels, mouse PSCs were pretreated with ARP water extract at various concentrations (50, 100, and 250 μg/ml) for 1 h, and then stimulated with PDGF-BB 25 ng/ml (315-18; PeproTech, Cranbury, NJ, United States) or TGF-β 0.5 ng/ml (7666-MB; R&D Systems Inc. Minneapolis, MN, United States) for 24 h. And TGF-β (0.5 ng/ml) was treated for 15 min to assess TGF-β signaling. To detect protein expression, mouse PSCs were pretreated with ARP (250 μg/ml) or SIS3 (5 μM) for 1 h followed by TGF-β (0.5 ng/ml) for 30 min, and whole-cell lysates were harvested for further experiments.

### Western blot

PSC proteins were lysed with 1 × RIPA lysis buffer containing 1% protease inhibitor cocktail and 1% phosphatase inhibitor and incubated on ice. The samples were boiled for 5 min in 62.5 mM Tris-HCl buffer with a pH of 6.8, containing 2% Sodium dodecyl sulfate (SDS) (S1377.0500; Duchefa biochemie, Netherlands), 20% glycerol, and 10% 2-mercaptoethanol. Proteins were separated on 8 and 10% SDS-polyacrylamide gels and transferred to nitrocellulose membranes. The membrane was blocked with 5% skim milk (232,100; Becton-Dickinson and Co., United Kingdom) in phosphate buffered saline (PBS; CNP010-1000; CellNest, Hanam, KyungKiDo, South Korea) containing Tween 20 (PBST) for 1 h under RT, followed by incubation with phosphorylated Smad2/3 (1:500; cat. No. 8828S, Cell Signaling Technology, Danvers, Massachusetts, United States), Smad2/3 (1:500; cat. No. 3102S; Cell Signaling Technology, Danvers, Massachusetts, USA), α-SMA (1:1000; cat. No. ab5694; Abcam, Cambridge, United Kingdom), GAPDH (1:1000; cat. No. 2118S; Cell Signaling Technology, Danvers, Massachusetts, United States), and Actin (1:1000; cat. No. 8457S; Cell Signaling Technology, Danvers, Massachusetts, United States) overnight at 4°C. After washing three times, the membrane was incubated with secondary antibodies for 1 h at RT. The proteins were visualized using an enhanced chemiluminescence detection system (Amersham, Buckinghamshire, United Kingdom), according to the manufacturer’s protocol.

### High-performance liquid chromatography analysis of *A. pericarpium* extract

To prepare a sample using the extract, 10 mg of water extract of ARP was dissolved in 1 ml distilled water and then filtered through a 0.45 μm syringe filter. Reference compounds such as (+)-catechin (99% purity, Cat. C1251) and 4-hydroxybenzoic acid (99% purity, Cat. H20059) were purchased from Sigma-Aldrich Chemical Co. The concentrations of the compounds were prepared as 0.125, 0.25, and 0.5 mg/ml in methanol for quantitative and qualitative analysis. High-performance liquid chromatography (HPLC) separation was performed using a YL-9100 series HPLC instrument equipped with a sample injector and UV/V detector (YoungLin. Republic of Korea). A YMC ODS-pack column (10 mm × 150 mm I. D., 5 μm, Japan) was used for separation. The ARP water extract sample (10 mg/ml) was injected at 20 μl. Subsequently, 10 μl of each compound was injected, and all sample injections were performed three times. For the HPLC analysis, 0.1% formic acid added to water (A)/acetonitrile(B) was used as the mobile phase with a gradient elution method: 0–10 min, an isocratic 5% B; 10–40 min, gradient from 5% B to 15% B; 40–55 min, gradient from 15% B to 100% B; 55–60 min held at 100% B. Flow rate was 0.7 ml/min, and detection wavelength was 254 and 280 nm.

### Statistical analysis

Results are expressed as mean ± standard error of the mean (SEM). Significance was evaluated using a two-way analysis of variance (ANOVA) with time and dose parameters. Significant ANOVA tests were further examined by post-hoc analysis using the Duncan method for multiple comparisons among groups. Values of *p* < 0.05 were accepted as statistically significant.

## Results

### Effects of *A. pericarpium* on biochemical parameters in mice

To determine an efficient concentration of the ARP water extract that is non-toxic with maximum biological efficacy, we examined biochemical parameters under normal conditions in mice. Mice were intraperitoneally administered either ARP or saline (control), and serum samples were harvested after 24 h for examination of biochemical factors. Compared to saline treatment, the administration of ARP did not lead to toxicity, even at concentrations below 400 mg/kg (Table 1). Based on human equivalent dose (HED) calculations, a concentration over 400 mg/kg is not realistic in clinical studies because of the large amount of drug administered. Therefore, we used ARP at concentrations below 400 mg/kg in the subsequent experiments to investigate its beneficial effects on CP.

**TABLE 1:** Biochemical parameters of mice with ARP water extract in acute toxicity test.

Group (mg/kg)	ALP (IU/L)	ALT (IU/L)	AST (IU/L)	BUN (mg/dl)<	CREA (mg/dl)
Saline	268.00 ± 30.35	24.33 ± 6.11	119.33 ± 37.54	21.00 ± 7.00	0.17 ± 0.03
ARP 10	247.00 ± 44.24	24.00 ± 2.65	133.00 ± 13.00	25.33 ± 4.93	0.17 ± 0.02
ARP 100	236.00 ± 19.08	24.33 ± 1.53	141.67 ± 32.87	21.00 ± 5.57	0.14 ± 0.03
ARP 200	238.33 ± 9.87	25.00 ± 4.58	124.00 ± 22.63	20.00 ± 2.65	0.15 ± 0.01
ARP 300	191.67 ± 43.84	24.00 ± 2.00	132.00 ± 26.91	20.67 ± 3.51	0.15 ± 0.01
ARP 400	231.33 ± 20.98	40.00 ± 16.37	142.33 ± 30.53	21.00 ± 3.61	0.17 ± 0.01

### Effects of *A. pericarpium* on morphological damage in cerulein-induced chronic pancreatitis

It is suggested that pancreatic acinar cell death occurs in the early stage of CP, and repeatedly damaged pancreatic acinar cell atrophy causes morphological changes such as pancreas size and duct-like structure, resulting in chronic inflammation (Kazacos and Van Vleet, 1989; Hashimoto et al., 2000; Su et al., 2001). To evaluate whether the ARP water extract can prevent the onset of CP, the pancreatic weight/body weight (PW/BW) ratio and pancreatic morphological changes were investigated in the cerulein-induced CP model. As anticipated, the proportion of PW/BW in the CP group was significantly lower than that in the normal group due to atrophy of the pancreatic mass. However, the decrease in the proportion of PW/BW significantly increased in the ARP treatment group (Figure 1B). Histological features of the pancreas in CP showed immune cell infiltration, acinar cell atrophy, and increased duct-like structures. In contrast, the ARP water extract-administered group showed a protective effect through significant improvement in glandular atrophy and inflammation in a dose-dependent manner (Figures 1C,D). In the determination of the effect of ARP on CP-induced morphological damages, treatment with 400 mg/kg of ARP resulted in an inhibitory activity similar to that observed with 200 mg/kg (data not shown). Thus, we used 200 mg/kg ARP as the highest concentration to exhibit anti-fibrogenic activity in CP.

### Effects of *A. pericarpium* on the activation of pancreatic stellate cells during chronic pancreatitis

**FIGURE 2:**
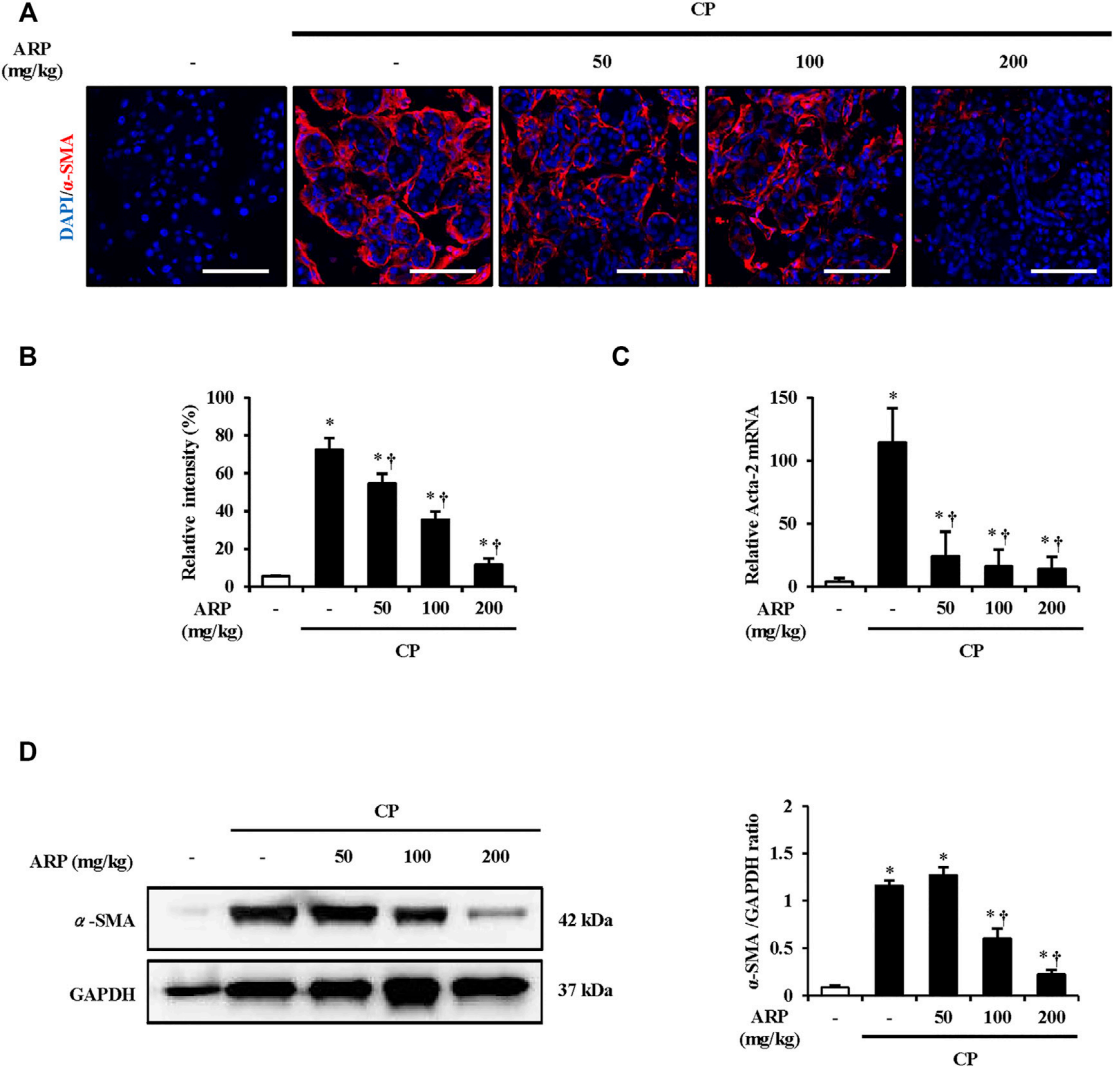
Effect of ARP water extract on PSC activation. **(A)** Confocal images of immunofluorescence staining for α-SMA in the saline, CP, and ARP groups (50, 100, and 200 mg/kg); the red color shows α-SMA positive cells, and the blue color shows DAPI positive cells. **(B)** The relative intensity of α-SMA. **(C)** Acta-2 mRNA level in the pancreas was assessed using RT-qPCR. **(D)** Protein expression of α-SMA was assess using western blot analysis and GAPDH was used as a loading control. Data are represented as means ± SEM (*n* = 3 per group). Results are representative of three experiments. *p < 0.05 vs saline alone; ^†^ *p* < 0.05 vs CP. Original magnification, ×400. Scale bar = 50 μm.

When the pancreas is subjected to repeated injury, quiescent PSCs transform into a myofibroblast-like phenotypes and become activated and later synthesize and secrete fibrosis factors. The secretion of α-SMA is considered a major indicator of PSC activation (Apte et al., 2011). To determine whether the ARP water extract regulates PSC activation in CP, Immunofluorescence (IF) staining was performed. As shown in Figures 2A,B and Supplementary Figure S2A, while the number of cells stained with α-SMA was remarkably increased in CP tissues, the number was significantly reduced in tissues pretreated with AR.

P water extract. To clarify this, we also measured the mRNA level of Acta-2 and protein expression of α-SMA. The mRNA level of Acta-2 and the protein expression of α-SMA in CP were considerably increased, and it was well suppressed in the group pretreated with the ARP water extract (Figures 2C,D).

### Effects of *A. pericarpium* on the production of ECM components during chronic pancreatitis

**FIGURE 3:**
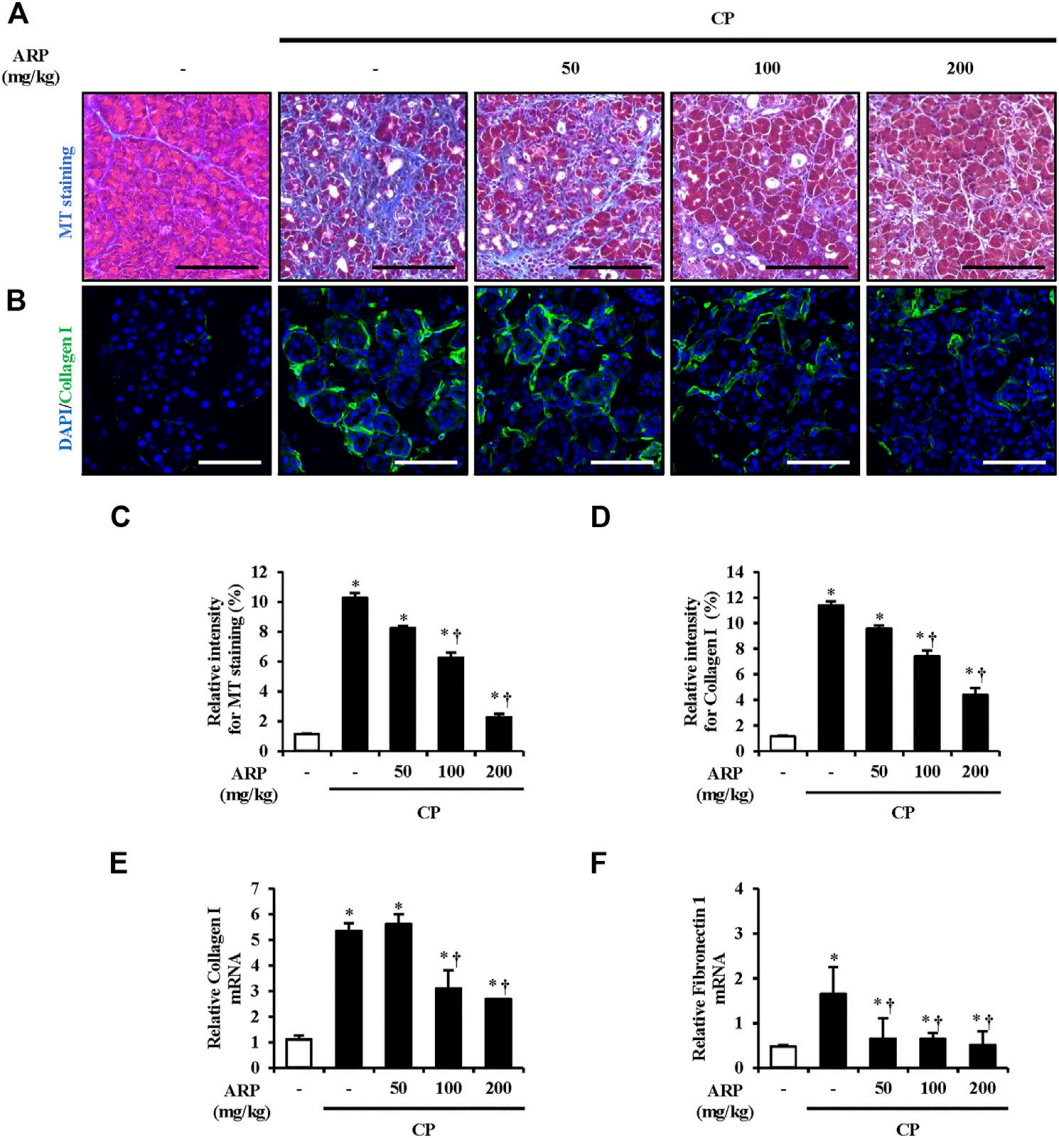
Effect of ARP water extract on ECM deposition during CP. **(A)** Masson’s trichrome staining for collagen deposition in the saline, CP, and ARP groups (50, 100, and 200 mg/kg). **(B)** Confocal images of immunofluorescence staining for collagen I in the saline, CP, and ARP groups; the green color shows collagen I positive cells, and the blue color shows DAPI positive cells. **(C)** The relative intensity of Masson’s trichrome staining. **(D)** Relative intensity of collagen I. **(E)** Collagen I mRNA level in the pancreas was assessed using RT-qPCR. **(F)** Fibronectin one mRNA level in the pancreas was assessed using RT-qPCR. Data are represented as means ± SEM (*n* = 3 per group). Results are representative of three experiments. *p < 0.05 vs. saline alone; ^†^ *p* < 0.05 vs. CP. Original magnification, ×400. Scale bar = 50 μm.

During fibrogenesis, secretion and production of ECM components are the main processes. Collagen, which is the most remarkable feature, and fibronectin, which occurs before collagen deposition and regulates the accumulation of collagen, are important factors that must be assessed in this process (Velling et al., 2002; Sottile et al., 2007; To and Midwood, 2011). To assess the preventive effect of the ARP water extract on collagen deposition, MT and IF staining were performed. As shown in Figures 3A–D and Supplementary Figure S2A, collagen deposition and the areas of collagen I staining (green) were substantially increased in CP tissue. However, overall collagen deposition in both MT and IF staining was noticeably decreased in the pancreas pretreated with the ARP water extract. We also examined mRNA levels of collagen I and FN1. Similar to the results of staining, the mRNA levels of collagen I and FN1, which were upregulated in CP, were significantly inhibited in the group pretreated with ARP water extract (Figures 3E,F).

### Effects of *A. pericarpium* on the pancreatic stellate cells activation in the isolated pancreatic stellate cells

**FIGURE 4:**
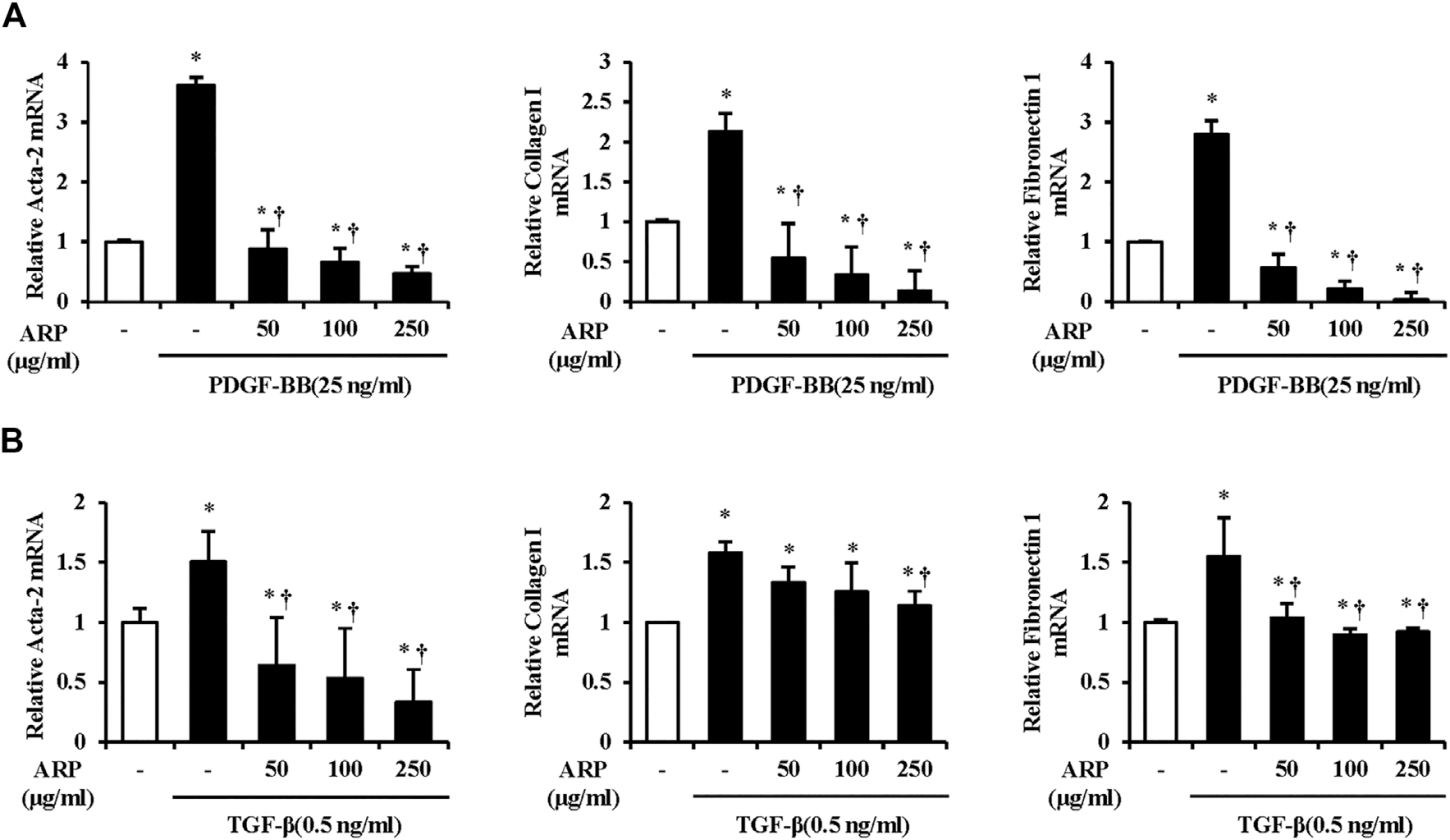
Effect of ARP water extract on PSC activation and ECM production on the isolated PSCs. **(A)** PDGF-BB induced Acta-2, collagen I, and fibronectin one mRNA levels in isolated PSCs were assessed using RT-qPCR. **(B)** TGF-β induced Acta-2, collagen I, and fibronectin one mRNA levels in isolated PSCs were assessed using RT-qPCR. Data are represented as means ± SEM. Results are representative of three experiments. *p < 0.05 vs. distilled water alone; ^†^ *p* < 0.05 vs. PDGF-BB or TGF-β1.

The most prominent feature of CP is fibrosis, and activated PSCs are known to play a pivotal role in the production of ECM components in this process (Ignotz and Massague, 1986; Bachem et al., 1998). To assess the effect of ARP water extract on the PSC activation, we measured the mRNA levels of Acta-2, collagen I, and FN1 in isolated PSCs. As shown in Figures 4A,B, the mRNA levels of Acta-2, collagen I, and FN1 were increased in PDGF or TGF-β treated groups compared with control group. However, ARP water extract decreased the mRNA levels of increased Acta-2, collagen I, and FN1, which were upregulated by PDGF or TGF-β.

### The Mechanism of *A. pericarpium* to regulate pancreatic stellate cells activation in the isolated pancreatic stellate cells

**FIGURE 5:**
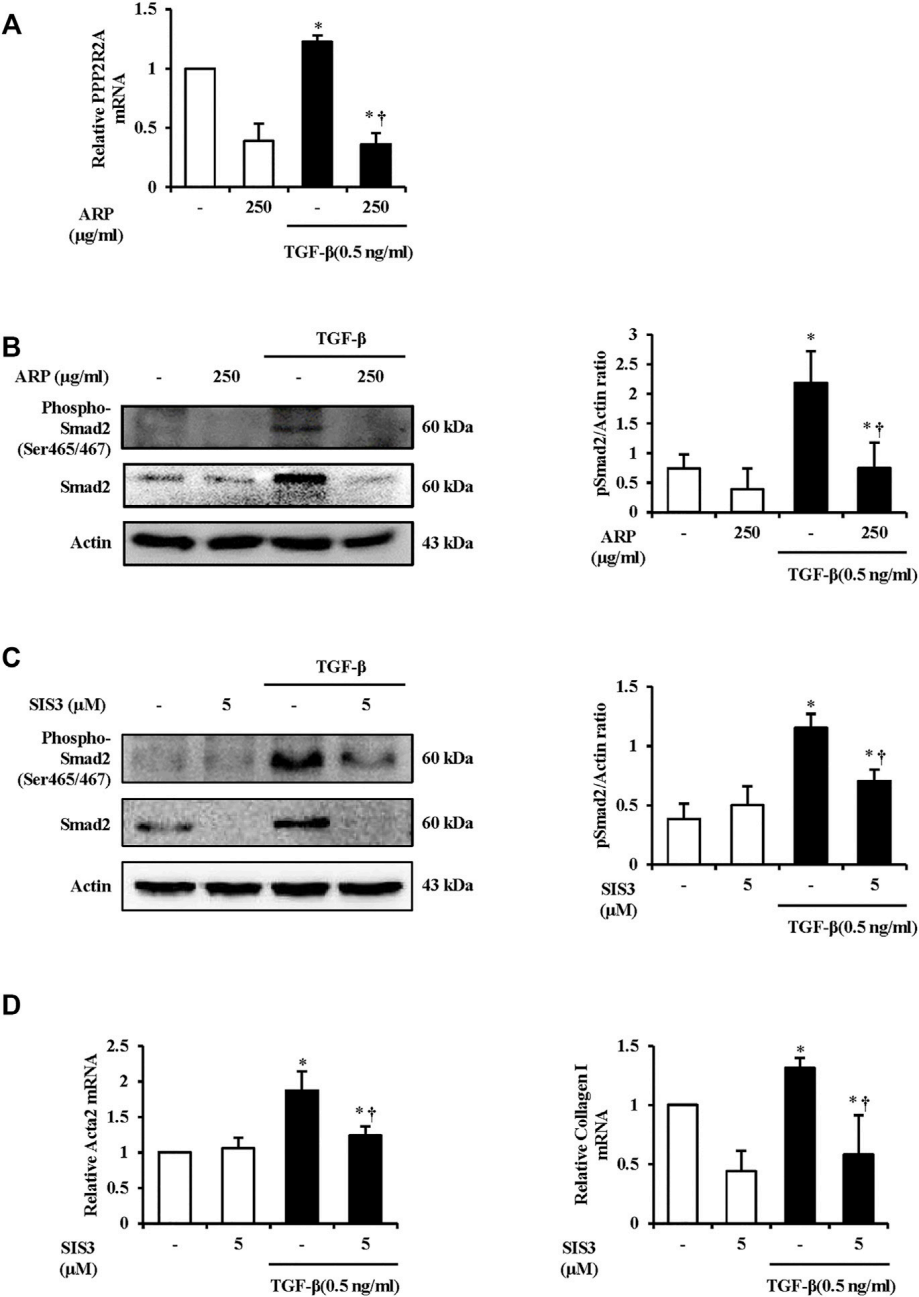
The Mechanism of ARP to Regulate PSC Activation in the Isolated PSCs. **(A)** TGF-β induced PPP2R2A mRNA levels in isolated PSCs were assessed using RT-qPCR. **(B,C)** Protein expression of phosphorylated Smad2 (Ser465/467) was assess using western blot analysis and Actin was used as a loading control. **(D)** TGF-β induced Acta2 and collagen Ⅰ mRNA levels in isolated PSCs were assessed using RT-qPCR. Data are represented as means ± SEM. Results are representative of three experiments. *p < 0.05 vs. distilled water alone; ^†^ *p* < 0.05 vs. TGF-β1.

To investigate how ARP water extract regulates PSC activation, we first measured the mRNA level of PPP2R2A. We found that the mRNA level of PPP2R2A was upregulated after TGF-β stimulation. On the other hands, levels were reduced in the group that treated ARP water extract (Figure 5A). And we checked the protein expression of Smad2. Compared with the control, Smad2 phosphorylation occurred in PSCs treated with TGF-β and in the presence of ARP water extract, phosphorylated Smad2 was remarkably suppressed (Figure 5B). To confirm that ARP inhibits PSC activation through Smad2, we further conducted experiments by pretreating SIS3, inhibitor of Receptor activated Smads (R-Smads) (Miyake et al., 2020). As shown in Figure 5C, the increased Samd2 due to TGF-β was effectively reduced in the SIS3 treatment group. Moreover, SIS3 reduced the mRNA levels of Acta-2 and collagen I induced by TGF-β compared to the TGF-β only treatment group (Figure 5D).

### Therapeutic effects of *A. pericarpium* during chronic pancreatitis

**FIGURE 6:**
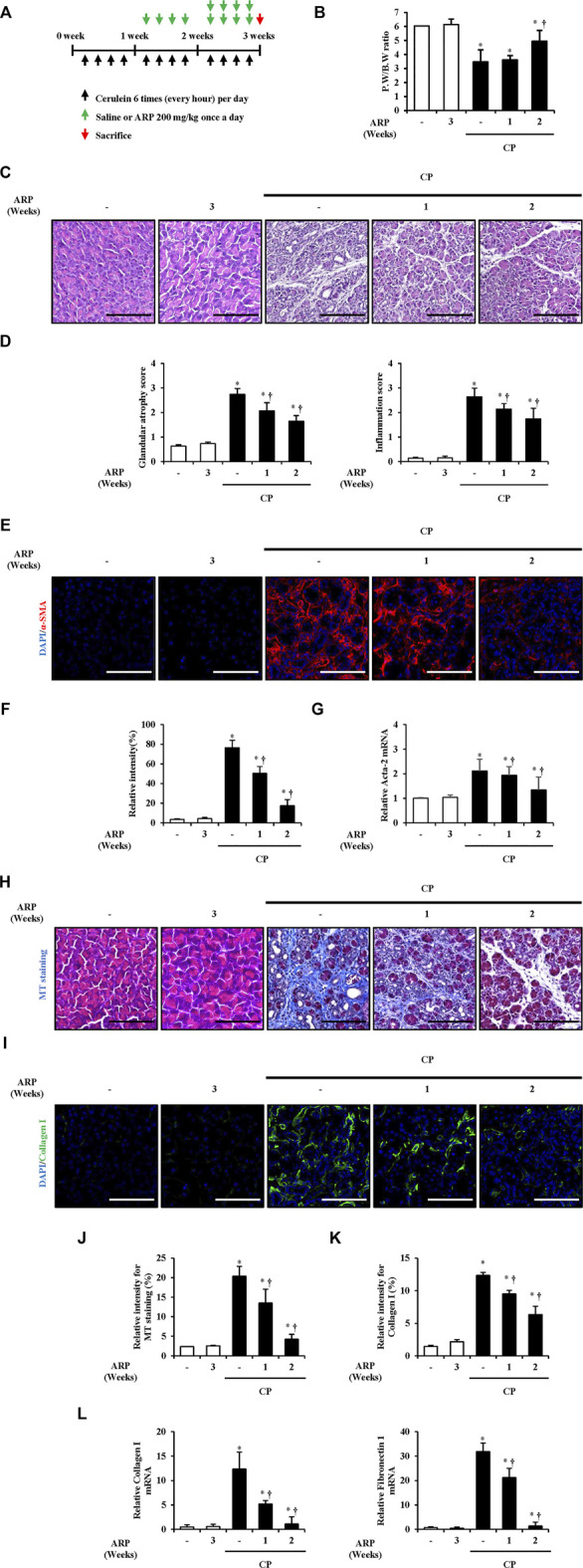
(Continued). Therapeutic effects of ARP water extract during CP. **(A)** Scheme of CP model in mice to see the therapeutic effect of ARP water extract. **(B)** Pancreatic weight/body weight ratio in the saline, ARP alone, CP and post-treated-ARP groups (200 mg/kg). **(C)** Representative Hematoxylin and Eosin-stained sections of the pancreas. **(D)** Pathological scores of pancreatic tissues. **(E)** Confocal images of immunofluorescence staining for α-SMA in the saline, ARP alone, CP and ARP groups (200 mg/kg); the red color shows α-SMA positive cells, and the blue color shows DAPI positive cells. **(F)** Relative intensity of α-SMA **(G)** Acta-2 mRNA level in the pancreas was assessed using RT-qPCR. **(H)** Masson’s trichrome staining for collagen deposition in the saline, ARP alone, CP and ARP groups (200 mg/kg). **(I)** Confocal images of immunofluorescence staining for collagen Ⅰ in the saline, ARP alone, CP and ARP groups; the green color shows collagen I positive cells, and the blue color shows DAPI positive cells. **(J)** Relative intensity of Masson’s trichrome staining. **(K)** Relative intensity of collagen I. **(L)** mRNA levels of collagen Ⅰ and fibronectin one in the pancreas were assessed using RT-qPCR. Data are represented as means ± SEM (*n* = 3 per group). Results are representative of three experiments. *p < 0.05 vs saline alone; ^†^ *p* < 0.05 vs. CP. Original magnification, ×400. Scale bar = 50 μm. .

Based on the above results, we investigated whether ARP water extract has a therapeutic effect on CP. We designed an experiment to inject ARP intraperitoneally 2 weeks or 1 week after the onset of CP so as to administer ARP for 1 week or 2 weeks (Figure 6A). As a result, pancreatic injury, including glandular atrophy, destruction, and inflammatory cell infiltration, was noticeably alleviated by post-treatment with ARP during CP (Figures 6B–D). Moreover, α-SMA expression and collagen deposition were gradually decreased by ARP during CP. And the elevated Acta-2, collagen I, and FN1 levels were repressed when post-treated with ARP, indicating that ARP could exhibit therapeutic effects in CP (Figures 6E–L and Supplementary Figure S2B).

### Analysis components isolated from *A. pericarpium* extracts

**FIGURE 7:**
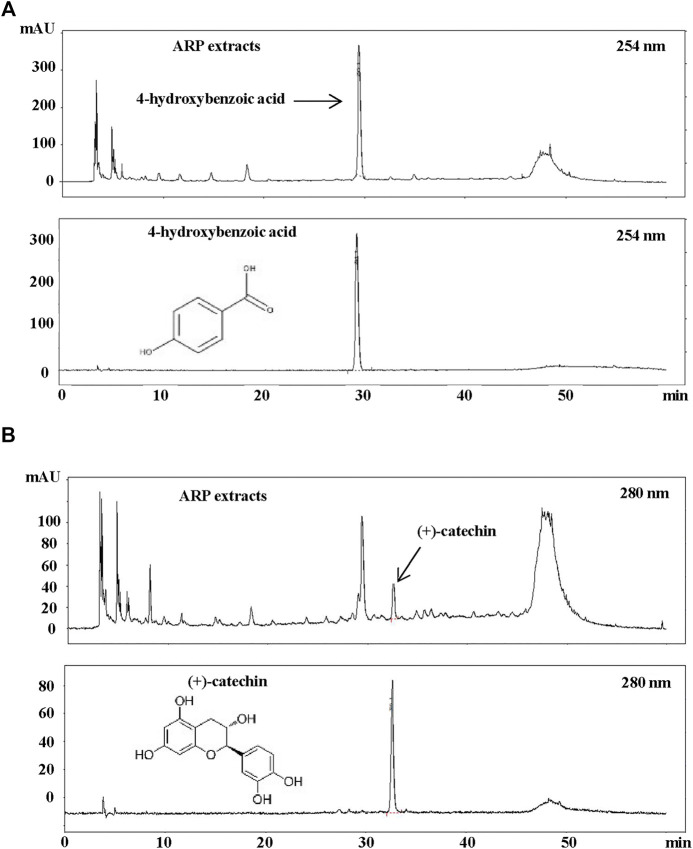
HPLC Analysis of ARP water extracts. ARP extract was dissolved in distilled water and filtered through a 0.45 μm syringe filter. As standard, methanol-dissolved 4-hydroxybenzoic acid and (+)-catechin (99% purity) were used. A sample volume of 20 μl was injected and analyzed at a flow rate of 0.7 ml/min using an ultraviolet detector at **(A)** 254 nm for 4-hydroxybenzoic acid and **(B)** 280 nm for (+)-catechin.

Few studies have been conducted on the chemical profile of ARP. Most studies have focused on alkaloids, such as arecoline and guvacoline, associated with Arecae Semen, the seed of ARP-enclosed fruit (Yu et al., 2016; Tian et al., 2018). Thus, to determine which active components are involved in the ARP activity, optimized HPLC was conducted using the ARP water extract based on a previous report (Huang et al., 2010). As shown in Figure 7, analysis by HPLC of ARP water extract provided major peaks at 29.6 and 33.7 min, and these peaks were identified as 4-hydroxybenzoic acid (Figure 7A) and (+)-catechin (Figure 7B) based on the comparison of the retention time in chromatograms of compounds. We also calculated the concentrations of these two components and found that ARP water extract contained approximately 0.604% of 4-hydroxybenzoic acid and 0.388% of (+)-catechin.

## Discussion

Sequential pancreatic damage occurs due to repeated inflammation, resulting in increased pancreatic fibrotic tissue (Beyer et al., 2020). Pancreatic damage first appears as vacuoles and acinar cell death. Simultaneously, as the damaged pancreatic acini atrophy, cytoplasmic vacuolization worsens, the number of fibroblasts between the tissues and collagen deposition increases, leading to so-called CP (Kazacos and Van Vleet, 1989). According to a recent study, it was confirmed that the size of pancreas in CP patients decreased by nearly 20%, and in particular, the incidence of apoptosis in acinar cells increased by 10 times compared to that in the normal group (Schrader et al., 2009). It has also been reported that the histological and inflammatory score of CP worsens as the expression of factors that cause apoptosis of acinar cells increases (Singh et al., 2008; Li et al., 2021). Therefore, the inhibitory effect of glandular destruction is considered an important factor in improving CP. In our study, glandular destruction occurred in the case of CP induced after repeated injections of cerulein. However, when the ARP water extract was pretreated, it was found that pancreatic parenchyma destruction, including acini atrophy and inflammation, decreased in a dose-dependent manner (Figure 1).

CP is affected by injured acinar cells and inflammatory cells, as it is caused by repeated pancreatic damage, and PSC activation is caused by factors such as cytokines and growth factors produced by these cells. If activated PSC continues to be stimulated by exogenous or endogenous factors, permanent pancreatic fibrosis occurs. α-SMA is then expressed in the fibrotic tissue area replacing the normal parenchymal tissue in which PSC is activated, and the synthesis of ECM factors, including diverse types of collagen and fibronectin, increases in this area (Schneider et al., 2001; Krieg et al., 2007; Apte et al., 2011). Thus, we examined whether ARP reduces the above-mentioned factors which are increased in CP, as reported previously (Barnes et al., 1999; Bachem et al., 2006; Chiang et al., 2009). As shown in Figures 2, 3, ARP prevents PSC activation and ECM production by effectively reducing the expression of α-SMA and collagen deposition which occurs during CP progression.

The continuous incidence of pancreatic injuries and inflammation results in the repetitive activation of PSCs, which are originally in a quiescent state and eventually undergo pancreatic fibrosis (Masamune et al., 2009). Regulating PSCs is an important factor in treating pancreatic fibrosis in which PSCs are activated through both paracrine and autocrine loops (Apte et al., 1999; Mews et al., 2002; Ohnishi et al., 2003; Masamune et al., 2009). When PSCs are transformed into a myofibroblast-like phenotype, Ca^2+^ signaling, known to increase in the early stages of AP, appears remarkably. PDGF is believed to be involved in PSC proliferation through Ca^2+^ signals, as the intracellular Ca^2+^ release and PDGF receptors in fibrotic areas increase when cells are exposed to PDGF (Pinzani et al., 1991; Apte et al., 1999; Won et al., 2011; Pallagi et al., 2020). Studies have shown that PDGF regulates the number of cells and DNA synthesis in PSCs (Apte et al., 1999; Luttenberger et al., 2000). TGF-β greatly contributes to irreversible pancreatic fibrosis by expressing α-SMA in PSCs, increasing PDGF receptors, and ECM protein synthesis (Ignotz and Massague, 1986; Apte et al., 1999). Additionally, it has been reported that the concentration of ECM components decreases when CP is induced under TGF-β inhibitory conditions, indicating that TGF-β regulates fibrosis (Menke et al., 1997; Luttenberger et al., 2000). Because of these characteristics, PDGF is used as a stimulator of PSC proliferation, and TGF-β is used as the main stimulator of PSC activation (Luttenberger et al., 2000). As the ARP water extract effectively inhibited the mRNA levels of Acta-2, collagen I, and FN1 in PSCs with PDGF or TGF-β exposure, we speculated that ARP could control the population and activation of PSCs (Figures 4A,B).

TGF-β receptor (TGF-βR) is a kinase that controls the TGF-β signaling, and it also serves as a substrate for their activity (Huang and Chen, 2012). The activated TGF-βR takes part in fibrosis by controlling TGF-β downstream signaling molecules such as mitogen activated protein kinases (MAPKs), Akt, TAK1 as well as Samds phosphorylation (Biernacka et al., 2011; Huang and Chen, 2012; Fan et al., 2020; Chung et al., 2021). In particular, TGF-βR1, containing ALK4/5, regulates the phosphorylation Smad2 and also contributes to fibrogenesis in CP (Fan et al., 2020; di Mola et al., 1999; Feng and Derynck, 2005). PPP2R2A, regulatory subunit of the PP2A, accelerates TGF-β/Activin/Nodal signaling by stabilizing the basal level of ALK4/5 and keeping them from degrading (Batut et al., 2008). Moreover, PPP2R2A indirectly promotes Smad2 phosphorylation and nuclear translocation of Smad2 in response to TGF-β (Batut et al., 2008). We found that mRNA level of PPP2R2A was higher in TGF-β treated PSCs and lower in ARP-treated PSCs (Figure 5A). This indicates that possibility of direct or indirect participation in the phosphorylation process of Samd2 by affecting TGF-βR1. However, not much research has been conducted on pancreatitis, so further research is needed to confirm it.

Smad2, which is primarily involved in PSC activation, has C-terminal SXS motif with serines that are directly phosphorylated by TGF-βR1 (Ohnishi et al., 2004; Feng and Derynck, 2005). When the TGF-βR1 induces phosphorylation of serines of the C-terminal SXS (Ser465/467), it is separated from the receptor and forms heterotrimer in which one common Smad (Co-Smad) and two R-Smads are combined. Then it translocates into the nucleus, and plays a role in the synthesis of profibrotic mediators and ECM proteins (Derynck and Zhang, 2003; Feng and Derynck, 2005; Lee et al., 2014). Therefore, Smads are present as downstream molecules of TGF-β and serve as modulators of TGF-β (Shimizu, 2008). In particular, the TGF-β1/Smad2 signaling in CP needs to be noted in that α-SMA protein expression in Smad2 overexpressed PSCs was higher than that of Smad3 (Ohnishi et al., 2004). Aside from this, live fibrosis was regulated through TGF-β1/Smad2 signaling in stellate cells (Lin et al., 2018). In our research, ARP water extract and SIS3 effectively inhibited protein expression of phospho-Smad2 and Smad2 (Ser465/467) in PSCs activated with TGF-β (Figures 5B,C). Referring to the corresponding data, we pretreated SIS3 and assessed Acta2 and collagen Ⅰ at the mRNA level to confirm that ARP water extract regulates PSC activation *via* TGF-β1/Smad2. Similar to the results of ARP water extract, SIS3 inhibited upregulation of the mRNA levels of Acta2 and collagen Ⅰ in PSCs treated with TGF-β (Figure 5D). Therefore, we could draw the conclusion that ARP inhibited PSC activation and ECM production by targeting the TGF-β/Smad2 axis.

To date, pancreatitis treatments mainly involve taking medications, surgical treatment, and implementing lifestyle changes, such as quitting smoking and stopping alcohol consumption (Singh et al., 2019). Primary medication for the treatment of CP involves acetaminophen, anti-inflammatory analgesic agents, or pancreatic enzyme supplements (Jadad and Browman, 1995; Burton et al., 2011; Singh et al., 2019). However, analgesics are at risk of side effects for long-term use, and pancreatic enzyme replacement therapy, a representative method of non-analgesic therapy, has limited effectiveness. Moreover, the efficacy of antioxidant agents has conflicting perspectives (Bhardwaj et al., 2009; Burton et al., 2011; Siriwardena et al., 2012; Olesen et al., 2013). Therefore, there is an urgent need to develop an effective treatment for CP, with fewer side effects. While the inhibitor used in the study has not yet been confirmed to be safe in clinical practice (Brenneman and Iyer, 2020), clinical studies have shown that there were no specific side effects when take a prescription which contains ARP for about a month (Liu et al., 2022). Thus, in this study, we examined whether ARP water extract alleviates CP severity during CP progression (Figure 6). As a result, the therapeutic effect of ARP in CP was confirmed by effectively inhibiting pancreatic damage, PSC activation and ECM production when ARP was injected for 1 week and 2 weeks. Taken together, we need to continue further experiments, including safety and efficacy, by referring to clinical trials using existing natural product (Paller et al., 2016), focusing on the CP improvement effect of ARP.

We found 4-hydroxybenzoic acid and (+)-catechin in the ARP water extract (Figure 7). Although the beneficial activity of 4-hydroxybenzoic acid in fibrosis has been rarely reported, (+)-catechin has been reported to exhibit strong anti-oxidant and anti-fibrotic activity (Yoo et al., 2005; Braicu et al., 2013). Thus, among the components of ARP, (+)-catechin is anticipated to have a beneficial effect on CP. In addition, studies have shown that catechin-related structures suppress ethanol-induced activation and ECM production of PSC and inhibit inflammation and α-SMA expression in CCl_4_ induced rat fibrosis model (Asaumi et al., 2006; Braicu et al., 2013; Wang et al., 2019). Based on this, it is highly likely that (+)-catechin acts as an active component for producing an anti-fibrotic effect of ARP water extract; therefore, further research will be conducted to determine whether (+)-catechin has a CP improvement effect.

## Conclusion

Taken together, we demonstrated that ARP water extract inhibited pancreatic morphological damage. Additionally, treatment with ARP reduced fibrosis-related factors such as α-SMA, collagen I, and FN1 in CP. We also found that ARP protects against the progression of pancreatic fibrosis by blocking the TGF-β/Smad pathway. Our findings suggest that ARP could be a possible candidate to be a therapeutic agent for the treatment of CP. Moreover, the possible effects of the active components of ARP and their combinations should be further studied.

## Data Availability

The original contributions presented in the study are included in the article/Supplementary Materials, further inquiries can be directed to the corresponding authors.

## Supplementary material

SUPPLEMENTARY FIGURE S1 Melting curves of qRT-PCR. Melting curves for GAPDH, **(B)** Acta-2, **(C)** Collagen Ⅰ, **(D)** FN1 and **(E)** PPP2R2A.

SUPPLEMENTARY FIGURE S2 Immunofluorescence staining, showing co-expression of α-SMA and collagen Ⅰ. Confocal images of immunofluorescence staining for α-SMA and collagen Ⅰ in the saline, CP, and ARP groups (50, 100, and 200 mg/kg); the red color shows α-SMA positive cells, the green color shows collagen Ⅰ positive cells and blue color shows DAPI positive cells. **(B)** Confocal images of immunofluorescence staining for α-SMA and collagen Ⅰ in the saline, ARP alone, CP and ARP groups (200 mg/kg); the red color shows α-SMA positive cells, the green color shows collagen Ⅰ positive cells and blue color shows DAPI positive cells. Original magnification, x400. Scale bar = 50 μm.

## Abbreviations

ARP, Arecae pericarpium; Co-Smad, common Smad; CP, chronic pancreatitis; DAPI, 4′6-diamidno-2-phenylidole; ECM, extracellular matrix; FN1, fibronectin 1; HPLC, high-performance liquid chromatography; HPRT, hypoxanthine-guanine phosphoribosyl-transferase; IF, immunofluorescence; MAPK, mitogen activated protein kinases; MT staining, Masson’s trichrome staining; PDGF, platelet-derived growth factor; PSCs, pancreatic stellate cells; PW/BW, pancreatic weight/body weight; R-Smads, receptor activated Smads; RT, room temperature; TGF-β, transforming growth factor-β; TGF-βR, TGF-β receptor; α-SMA, alpha-smooth muscle actin.
