# Supplementary figures and images for: Arecae pericarpium water extract alleviates chronic pancreatitis by deactivating pancreatic stellate cells
Source: Front Pharmacol. 2022 Aug 29;13:941955. doi: 10.3389/fphar.2022.941955 (PMC9465814; doi:10.3389/fphar.2022.941955)

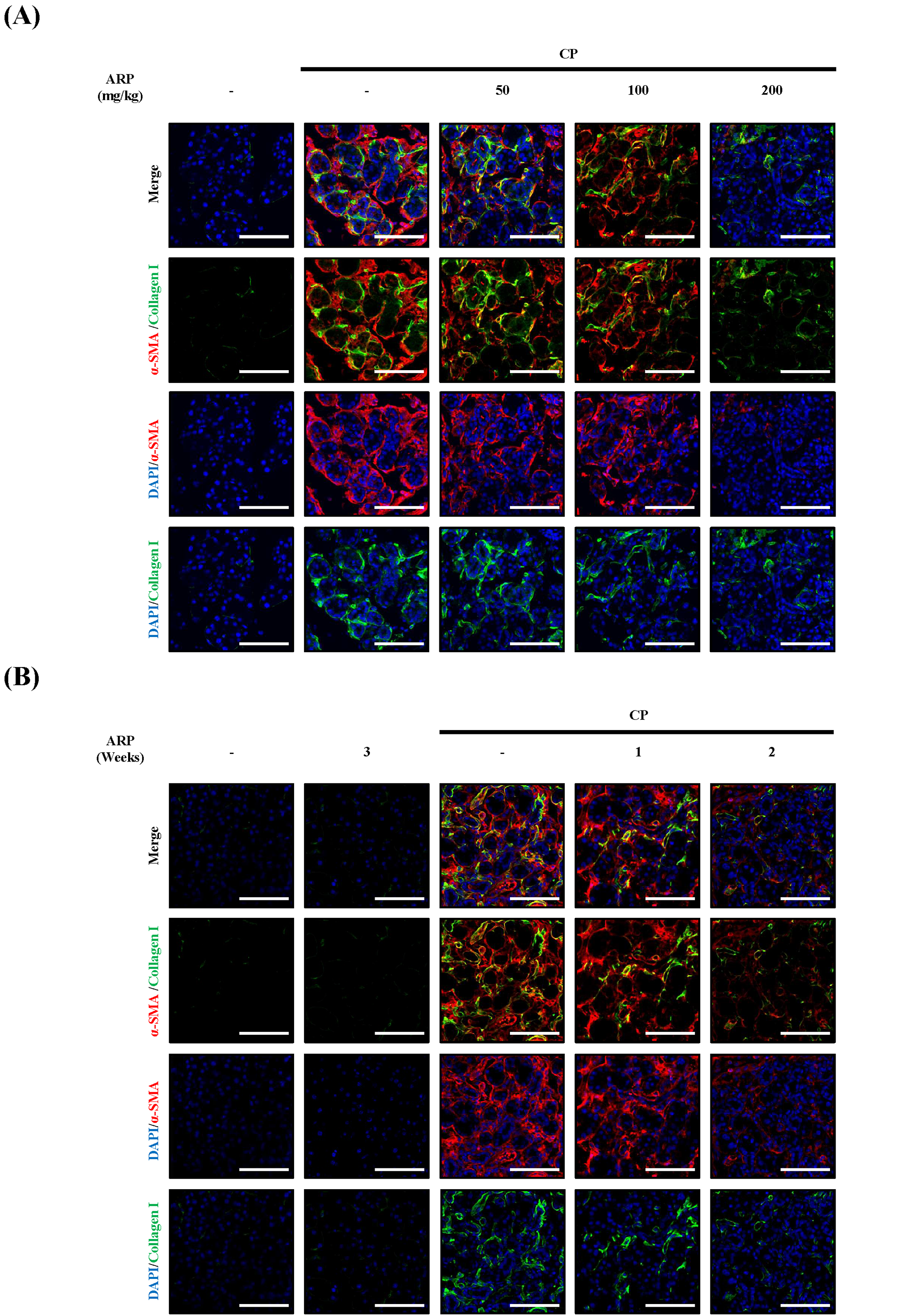

Supplement: Supplementary file 1 [file Image2.tif]

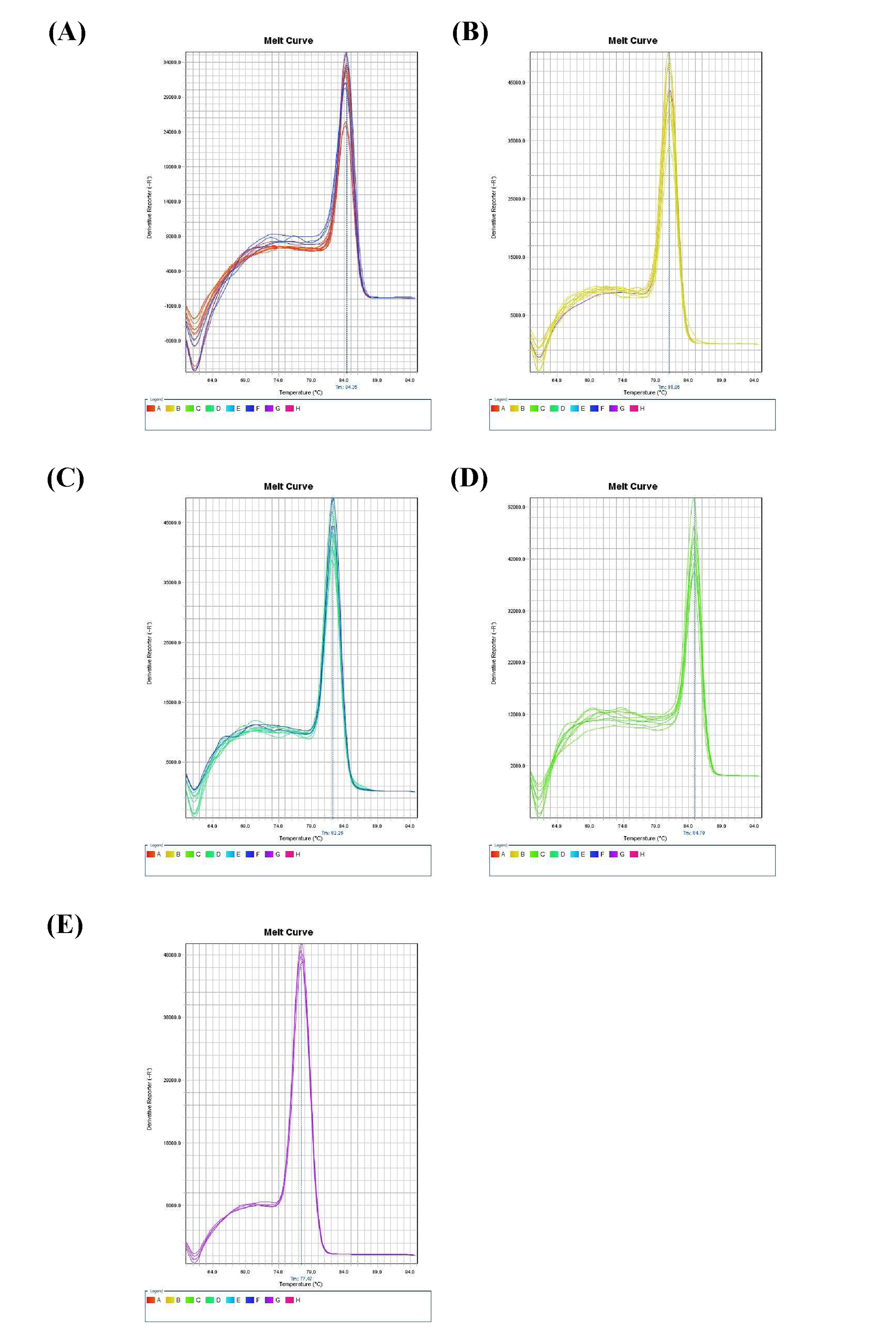

Supplement: Supplementary file 2 [file Image1.tif]
